# Freezability of Dog Semen after Collection in Field Conditions and Cooled Transport

**DOI:** 10.3390/ani12070816

**Published:** 2022-03-23

**Authors:** Martina Colombo, Maria Giorgia Morselli, Giulia Franchi, Sabine Schäfer-Somi, Gaia Cecilia Luvoni

**Affiliations:** 1 Dipartimento di Medicina Veterinaria e Scienze Animali (DIVAS), Università degli Studi di Milano, 26900 Lodi, Italy; mgiorgia.morselli82@gmail.com (M.G.M.); giulia.franchi.vet@hotmail.com (G.F.); cecilia.luvoni@unimi.it (G.C.L.); 2 Platform for Artificial Insemination and Embryo Transfer, Vetmeduni Vienna, 1210 Vienna, Austria; sabine.schaefer@vetmeduni.ac.at

**Keywords:** canine, chilling, cooling, cryopreservation, freezing, shipment, sperm, Uppsala

## Abstract

**Simple Summary:**

Dog semen freezing is gaining popularity, but it has to be performed in equipped facilities, which can be far from the place where the stud dog lives. To avoid animal movement, it seemed interesting to investigate whether freezing dog semen after 24 or 48 h of cooled transport to an equipped laboratory was possible when semen collection was performed in the field such as in local breeding kennels. The influence of two pre-freezing holding times (i.e., 24 or 48 h) and two holding diluents (solutions used to dilute semen before freezing) was evaluated. Post-thaw morphofunctional sperm features, such as motility, morphological integrity, and ability to bind female gametes, were assessed. No differences between times or diluents were observed, but motility tended to decrease in the samples frozen at 48 h. Since the insemination dose was based on the number of motile spermatozoa, a shorter pre-freezing time is advisable. Yet, considering that the rest of the morphofunctional parameters remained comparable between samples frozen after collection or after 24/48 h of transport, freezing after cooled transport is a good option for avoiding animal stress and for promoting a greater diffusion of semen cryopreservation.

**Abstract:**

Dog semen freezing is gaining popularity, but it has to be performed in equipped facilities, which can be far from the place where the stud dog lives. The aim of this study was to evaluate whether freezing dog semen after 24 or 48 h of cooled transport to an equipped laboratory was possible when semen collection was performed in the field such as in local breeding kennels. Single ejaculates from different dogs (mixed breeds and ages) were collected. In Experiment I, 10 ejaculates were conventionally frozen using the Uppsala method or frozen after 24 or 48 h of storage in a Styrofoam transport box cooled by icepacks. In Experiment II, 10 ejaculates were used to assess the influence of two extenders (Uppsala chilling extender or freezing extender 1) used for semen dilution during the 24 or 48 h storage. Motility, morphology, membrane, and acrosome integrity were analyzed as well as spermatozoa zona-binding ability. No significant differences were observed among the frozen groups, regardless of freezing time (Experiment I) or extender (Experiment II). Motility at thawing, however, decreased in absolute value at 48 h. Freezing of freshly collected semen is the gold standard, but the results obtained in this study prompt the application of freezing after cooled transport for the long-term preservation of dog semen, especially if the transport can be organized in 24 h.

## 1. Introduction

Thanks to the possibility of using frozen semen for artificial insemination in bitches with satisfactory pregnancy rates (45–70%, [1,2,3,4,5,6]), dog semen freezing is becoming increasingly popular among breeders and pet owners. Long-term stored spermatozoa are ready to be used in the optimal insemination time of the bitch, even if the animals live in different locations or cannot meet at the same time [7]. Frozen semen can also be useful for the preservation of genetic material of valuable subjects, such as those with specific phenotypic or work-related traits (e.g., champions of exhibitions and shows, elite working dogs with excellent abilities including assistance of disabled people, drug detection, and rescue activity), and for the preservation of genetic diversity, which is especially important for threatened breeds or species in biodiversity preservation programs [4,8]. Finally, the use of frozen semen can be exploited to optimize inseminating doses and speed up genetic selection, since more females can be inseminated with the same ejaculate [9], especially in farm species, or to reduce the spread of reproductive venereal disorders, thanks to the fact that direct contact between animals is avoided.

In dogs, semen freezing works efficiently, and most of the time it is performed according to the Uppsala method [10,11]. This method involves a two-step dilution (the first one at room temperature and the second one after semen chilling and just before freezing) with egg yolk-based extenders containing increasing concentration of cryoprotectants (glycerol, equex) and exposure to liquid nitrogen vapors before the semen straws are plunged into liquid nitrogen. Although simple and straightforward, the procedure requires trained personnel and specialized facilities, and most veterinary practices are not equipped to perform it on their own.

In field conditions, as in local breeding kennels or houses where private-owned dogs live, the lack of equipment and know-how makes freezing procedures unfeasible. The distance from specialized freezing facilities often causes dog breeders and owners to travel several hours to preserve the semen of their stud dogs, increasing the cost and causing stress to the animals. To limit animal movement and to widen the diffusion of semen cryopreservation, the ejaculate could be collected in field conditions and shipped to a freezing facility to be cryopreserved. For instance, local veterinarians could perform the collection, dilute the semen, and ship the cooled sample to be frozen at specialized centers.

Indeed, semen chilling is a simple and easily diffusible procedure that only involves semen dilution and preservation at cooled temperatures. On the market, some solutions for chilled transport of dog semen are available, and the most used consists of a Styrofoam box with separate housings for the sample and for two icepacks that keep the temperature cool. Currently, shipping across countries is feasible in 24–48 h, and in this transport box, spermatozoa would be viable upon arrival at the freezing facility.

Some studies have already investigated the effect of chilling prior to freezing, but they were all in laboratory or semi-laboratory settings, using a refrigerator for semen equilibration. Generally speaking, dog sperm cells can be frozen after 24–48 h of chilled storage without deleterious effects. Indeed, post-thaw motility, acrosome integrity, and plasma membrane integrity or morphology of ejaculated spermatozoa frozen after 1 or 2 days at 4 °C remained comparable to those obtained after conventional freezing performed right after semen collection [12,13]. Similar results were also obtained with epididymal dog spermatozoa stored at 5 °C for up to 4 days [14]. In addition, 24 h cold storage prior to freezing had little effect on the oxidative status of sperm cells: while the level of superoxide dismutase (SOD) decreased, no changes were reported in glutathione peroxidase (GPx) activity and total reactive oxygen species (tROS) levels [15]. One study assessed sperm quality after 2 h pre-equilibration in a refrigerator at 5 °C and subsequent storage in a transport box for chilled semen for up to 45 h [16], and it reported that samples frozen after 24 h of cold storage showed no differences in comparison with the control frozen right after collection [16]. However, quality parameters tended to decrease along time, and temperature fluctuations might play a role in this. What would happen in field conditions if the samples were put directly in a transport box, such as the one commonly used for shipping, without temperature control and previous cooling remains to be investigated.

Therefore, the aim of this study was to assess the freezability of dog-ejaculated spermatozoa after 24 or 48 h cooled storage in field conditions. The cooled storage was performed in a Minitube^®^ transport box as would happen in field conditions, where a fridge is not available, and samples need to be shipped to an equipped lab for freezing without a preliminary chilling step. In Experiment I, the effects of field chilling on spermatozoa quality (motility, morphology, membrane, and acrosome integrity and oocyte-binding ability) were evaluated compared to conventional freezing right after semen collection. In Experiment II, two extenders were compared for the dilution of spermatozoa in the field and the chilling before freezing. The ultimate goal would be to suggest to owners, breeders, and veterinarians a valuable option to cryopreserve dog semen without moving animals even if they live far from a freezing facility.

## 2. Materials and Methods

### 2.1. Chemicals and Reagents

All chemicals and reagents were purchased from Merck KGaA (Darmstadt, Germany), unless otherwise stated.

### 2.2. Animals, Spermatozoa Collection, and Experimental Design

Healthy and pubertal stud dogs (*n* = 20) of different breeds, aged between 2 and 13 years (4.6–60 kg body weight), were included in this study, which was performed between November 2018 and November 2020. In particular, semen collection and freezing were performed between November 2018 and January 2019, while samples were thawed and analyzed between February 2019 and November 2020.

**Figure 1:**
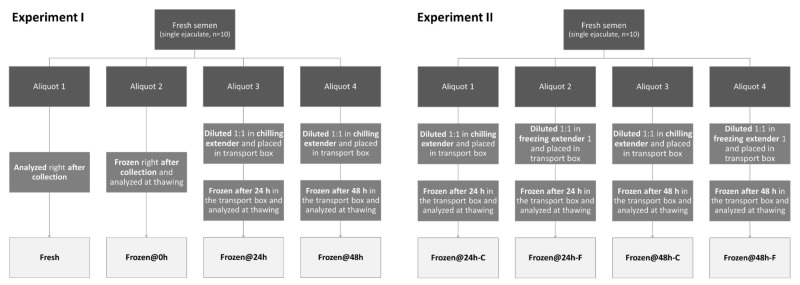
Experimental design.

Ejaculated spermatozoa were collected by digital manipulation (only once for each dog) and the sperm-rich fraction was used as fresh, cryopreserved right after collection, or cryopreserved after storage and transport in a Minitube^®^ transport box for chilled canine semen (Minitüb GmbH, Tiefenbach, Germany) as follows (Figure 1).

In Experiment I, collection (10 dogs) was performed in the veterinary teaching hospital, and semen was divided in 4 aliquots:

- One was analyzed at time zero, right after collection (Fresh);
- One was frozen at time zero, right after collection, and analyzed at thawing (Frozen@0h);
- One was diluted 1:1 in chilling extender [3], placed in the transport box, frozen after 24 h of storage, and analyzed at thawing (Frozen@24h);
- One was diluted 1:1 in chilling extender [3], placed in the transport box, frozen after 48 h of storage, and analyzed at thawing (Frozen@48h).

In Experiment I, icepacks were inserted in the transport box just before semen collection. The transport box was kept at room temperature until semen freezing.

In Experiment II, to evaluate the effect of holding extender and time on post-thaw sperm quality, collection (10 dogs) was performed in the field (i.e., dogs’ residences), and semen was divided in 4 aliquots in loco:

- One was diluted 1:1 in chilling extender [3], placed in the transport box, transported to the lab, frozen after 24 h of storage, and analyzed at thawing (Frozen@24h-C);
- One was diluted 1:1 in freezing extender 1 [3], placed in the transport box, transported to the lab, frozen after 24 h of storage, and analyzed at thawing (Frozen@24h-F);
- One was diluted 1:1 in chilling extender [3], placed in the transport box, transported to the lab, frozen after 48 h of storage, and analyzed at thawing (Frozen@48h-C);
- One was diluted 1:1 in freezing extender 1 [3], placed in the transport box, transported to the lab, frozen after 48 h of storage, and analyzed at thawing (Frozen@48h-F).

In Experiment II, icepacks were inserted in the transport box before leaving the lab to perform semen collection in the field (i.e., 1–2 h before). The transport box was kept at (winter) ambient temperature for transport, which occurred right after semen collection and dilution, while it was kept at room temperature after arrival at the lab.

Chilling extender was chosen because it is known to maintain semen features upon chilling, while freezing extender was chosen to mimic the first step of the Uppsala freezing, when semen is extended with freezing extender 1 and chilled, before being brought to the final volume with extender 2 and frozen [3].

In all of the samples and in both of the experiments, the motility, morphology, membrane integrity, acrosome status, and ability to interact with homologous oocytes by zona-binding assay were evaluated as explained below.

### 2.3. Semen Analysis

All the parameters were evaluated in fresh (Experiment I) and thawed samples (Experiments I and II). As detailed below, motility was also evaluated at two additional times.

#### 2.3.1. Motility

Motility was subjectively assessed on a pre-heated stage at 37 °C under a light microscope in fresh samples (pre-freezing motility of samples Frozen@0h), after transport just before freezing (pre-freezing motility for samples Frozen@24h and Frozen@48h) to determine the dilution factor for freezing of stored samples at a known concentration, after thawing, and 6 h after thawing to evaluate the survival time of thawed spermatozoa.

#### 2.3.2. Morphology

Sperm morphology was assessed in fresh and thawed samples applying the multiple-entry system as reported in the World Health Organization (WHO) guidelines (2010 and 2021) [17,18] and previously reported in the dog [19]. Briefly, spermatozoa were smeared on slides (two for each sample) and stained with Rose Bengal and Victoria Blue B. Two hundred spermatozoa for each sample were evaluated under a light microscope (Axiovert 100, Zeiss, Arese, Italy) with oil immersion objective at 100× magnification. Normal spermatozoa and all defect sites (i.e., head, neck, midpiece, tail, cytoplasmic droplets, and acrosome) in abnormal spermatozoa were recorded. Abnormal sperm heads included those that were pear-shaped, narrow at the base, detached, or with acrosome alteration (i.e., vesiculated or absent acrosome). Alterations of the neck/midpiece included a bent neck and proximal or distal cytoplasmic droplet, and an abnormal tail included a single bent, coiled, or broken tail. The calculation of the indices (multiple abnormalities index—MAI; teratozoospermic index—TZI; sperm deformity index—SDI) was performed as follows [17,18]:

MAI = (number of head defects + midpiece defects + tail defects)/total number of abnormal spermatozoa;

TZI = (number of spermatozoa with ≥1 head defect + number of spermatozoa with ≥1 midpiece defect + number of spermatozoa with ≥1 tail defect + number of spermatozoa with residual cytoplasmic droplets)/total number of abnormal spermatozoa;

SDI = (number of head defects + number of spermatozoa with ≥1 midpiece defect + number of spermatozoa with ≥1 tail defect + number of spermatozoa with residual cytoplasmic droplets)/total number of analyzed spermatozoa.

#### 2.3.3. Membrane Integrity and Acrosome Status

Membrane integrity and acrosome status were evaluated in fresh and thawed samples. Membrane integrity was assessed by the hypo-osmotic swelling test (HOS) as described for canine semen [20,21]. Briefly, an aliquot of semen (5 μL) was incubated in a pre-heated 150 mOsm hypotonic solution (0.735 g sodium citrate and 1.351 g fructose in 100 mL distilled water) at 37 °C and 5% CO_2_ for 30 min. Two hundred spermatozoa for each sample were evaluated under a light microscope with oil immersion and objective at 100× magnification and classified as curly (intact membranes) or not curly (altered membranes).

Acrosome status was evaluated by the peanut agglutinin (PNA) conjugated with fluorescein isothiocyanate (FITC) and propidium iodide (PI) staining under fluorescent microscope in at least 200 spermatozoa, according to the procedure described for stallion spermatozoa [22] and adapted to dog semen [19]. Acrosomes of stained spermatozoa were classified as intact (spermatozoa displaying intensively bright green fluorescence of the acrosomal cap, indicating an intact outer acrosomal membrane) or damaged (spermatozoa displaying disrupted fluorescence, fluorescent band at the equatorial segment, or no green fluorescence, indicating damages to the outer acrosomal membrane).

#### 2.3.4. Zona-Binding Assay (ZBA)

The ability of dog spermatozoa to interact with homologous oocytes was tested in fresh and thawed samples with an in vitro sperm function test, the ZBA. Canine frozen–stored ovaries [23] were warmed and sliced in phosphate-buffered saline (PBS) containing antibiotics, antimycotics, and 0.1% (*w*/*v*) polyvinyl alcohol (PVA) to release the oocytes. Only gametes with darkly pigmented ooplasm and intact zona pellucida (ZP, *n* = 756) were selected for the experiments and washed in canine capacitation medium (CCM, [7]) without antibiotics. Oocytes (*n* = 5–10 for each sample) were added to pre-warmed CCM droplets (10 μL) covered with mineral oil and incubated for equilibration in a controlled atmosphere (38.5 °C and 5% CO_2_ in air) for a minimum of 1 h. Thereafter, 40 μL of sperm suspension (0.5 × 10^6^ spermatozoa/mL) were added to each droplet, and oocytes and spermatozoa were co-incubated in a controlled atmosphere (38.5 °C and 5% CO_2_ in air) for at least 3 h. After incubation, the oocytes were gently washed in PBS/PVA to remove loosely attached spermatozoa, and sperm–oocyte complexes were stained with bisbenzimide (Hoechst 33342). The number of sperm bound to each ZP was counted under a fluorescent microscope [23,24]. Oocytes with ≥200 sperm bound were recorded as 200.

### 2.4. Semen-Freezing Procedure

Cryopreservation of spermatozoa was performed according to the Uppsala Equex-2 System [3,10] at a final concentration of 100,000 motile spermatozoa/µL. The pre-freezing concentration was determined by manual count with a Bürker chamber (data not shown). For samples Frozen@0h (Experiment I), the spermatozoa concentration was determined on the ejaculate, while for the samples Frozen@24h and Frozen@48h (Experiments I and II), the concentration was determined on samples diluted 1:1 and stored for 24/48 h in the transport box in order to calculate the dilution for freezing.

After collecting the sperm-rich fraction of the ejaculates and dividing it into aliquots and diluting it as described in the experimental design (chilling extender and freezing extender 1 were lab-made according to [3]), freezing was performed as follows.

For samples frozen right after collection (i.e., Frozen@0h), each sample was centrifuged (700× *g* for 5 min) to remove the supernatant, and spermatozoa were diluted in two steps (one at room temperature and one after cooling at 4 °C) with the freezing extender [3] (TRIS buffer with final concentrations of 5% glycerol, 1% Equex STM (Nova Chemical Sales Inc., Scituate, MA, USA), and 20% egg yolk). Samples diluted and stored for 24 or 48 h before freezing were centrifuged (700× *g* for 5 min) to remove the holding extender and the seminal plasma before dilution with the freezing extender in one single step.

When manipulating the chilled–stored samples (Frozen@24h and Frozen@48h in Experiment I and Frozen@24h-C, Frozen@48h-C, Frozen@24h-F, and Frozen@48h-F in Experiment II) all the equipment (i.e., tubes, pipette tips, and centrifuge) was kept at 4 °C.

For all the groups, after loading of the cooled samples in 0.5 mL straws, the straws were placed in a Styrofoam box in liquid nitrogen vapors (10 min, 4.5 cm above liquid nitrogen) and subsequently immersed into liquid nitrogen.

The straws were thawed in a water bath at 37 °C for 30 s.

### 2.5. Statistical Analysis

Data are shown as the mean ± standard deviation (SD). Statistical comparison between groups was performed using IBM SPSS statistics version 24 (Armonk, NY, USA). The Kolmogorov–Smirnov test was used for testing the normal distribution. In the case of normal distribution and after examination for homogeneity of variance, a mixed linear model (Sidak’s multiple comparison test) for comparison between groups was conducted. Data from Experiment I and Experiment II were analyzed separately. For each experiment, the treatment (i.e., Fresh, Frozen@0h, Frozen@24h, and Frozen@48h for Experiment I; Frozen@24h-C, Frozen@24h-F, Frozen@48h-C, and Frozen@48h-F for Experiment II) was chosen as the fixed parameters and all other related parameters as dependent variables. The dog was chosen as the subject variable. Subject variability (dog effect) was additionally analyzed using the Kruskal–Wallis test. Significance was set at *p* < 0.05.

## 3. Results

### 3.1. Experiment I

When comparing different holding times in the transport box, results showed that freezing after 24 or 48 h from semen collection did not affect post-thaw quality. Instead, as expected, the morphological quality of the fresh control was better than that of the frozen samples (*p* = 0.031, Table 1).

World Health Organization morphological indices increased after freezing (*p* = 0.031), regardless of freezing time, especially the SDI due to the abundance of acrosomal defects found during morphological evaluation (*p* < 0.0001). This trend was confirmed by FITC-PNA/PI intact acrosomes, which were significantly higher in fresh samples and lower in frozen ones (*p* = 0.001), although without differences between Frozen@0h, Frozen@24h, and Frozen@48h (*p* = 0.898). Similar data were obtained for membrane integrity (Fresh vs. Frozen: *p* = 0.018; *p* = 0.998 among different freezing times).

The results of motility are shown in Table 2. Although pre-freezing motility was lower after 48 h in the transport box (*p* = 0.017 vs. Frozen@0h) and numerical values tended to decrease with a longer pre-freezing holding time, no statistical differences were found in post-thaw motility among the frozen groups (*p* = 0.127).

Finally, the ability of spermatozoa to bind to the zona pellucida of frozen oocytes was not influenced neither by freezing nor by pre-freezing holding time in the transport box (Table 3). Specifically, the average number of spermatozoa bound to each oocyte was similar for all the groups, both when considering all the oocytes (*p* = 0.962) and when considered only the oocytes that had at least one bound spermatozoon (*p* = 0.911), which were also found in similar proportions in all the groups (*p* = 0.829).

### 3.2. Experiment II

The use of chilling or freezing extender to dilute and cool semen for 24 or 48 h in the transport box was comparable.

Morphology (Table 4) was not affected by extender type or freezing time. No differences were found in MAI, TZI, and SDI (*p* = 0.589), in FITC/PNA-PI intact acrosomes (*p* = 0.872), nor in membrane integrity (*p* = 0.895), which were consistent among extenders and timings.

Motility was the only parameter where significant differences were found (Table 5). Even though neither the extender nor the freezing time seemed to have an influence on pre-freezing motility (*p* = 0.676), motility at thawing sharply decreased at 48 h compared to 24 h when the chilling extender was used for dilution (*p* = 0.020), but it remained comparable with the results obtained at 48 h when the freezing extender was used for dilution (*p* = 0.780). After 6 h from thawing, motility was comparable in all the treatments (*p* = 0.204).

However, zona-binding ability was not influenced (Table 6), and the average number of bound spermatozoa per oocyte (*p* = 0.428) and per bound oocyte (*p* = 0.408) as well as the proportion of oocytes with bound spermatozoa (*p* = 0.437) were similar among all treatments.

In both the experiments, a dog effect was noticed, especially in Experiment II. Dogs were considered as subject variable, and the values of each of the parameters evaluated in the study were rarely overlapping among different individuals. This could explain the high standard deviations that characterized the results. In Experiment I, there was a significant effect for motility 6 h post thawing (*p* = 0.009) and a tendency towards significance (*p* = 0.06) for motility at thawing. In Experiment II, where only frozen–thawed groups were compared, besides motility pre-freezing (0.007) and post-thawing (*p* = 0.032), morphology was also significantly dependent on individuals (*p* = 0.002 for TZI and *p* < 0.001 for MAI and SDI). Therefore, in both the experiments, fresh semen quality was variable, which had an impact on semen quality at thawing. Nevertheless, the impact of storage time was clearly visible (Table 5).

## 4. Discussion

The possibility of freezing semen after storage and transport is a matter of great interest, both in veterinary and human medicine. Freezing requires to be performed in specialized facilities, and for semen donors living far away, it might be not convenient to reach a distant location. Some solutions should be investigated to cryopreserve semen samples when the collection is conducted somewhere else. For animals, such as dogs, most studies have focused on freezing after preservation at chilling temperatures up to 48 h [12,13,14,15]. In other species, such as the horse, semen can also be stored at 5 °C for some time before freezing, but only shorter holding times have been tested [25,26]. Similar freezing protocols would also be interesting for wild species, whose semen could be collected in the field and then shipped to a specialized laboratory for freezing and storage. Encouraging results have been obtained, for instance, in the brown bear, whose semen, after storage at 5 °C for 24–48 h and freezing, could give satisfactory post-thawing outcomes [27,28].

In humans, semen freezing has become popular because of the increasing use of medically assisted procreation. Men that are already involved in assisted procreation with their partners, or men that still cannot foresee their parenthood but want to preserve their fertility, often wish to cryopreserve their gametes, but they are not always available to go to a clinic for semen collection. For this purpose, several kits are already available on the market for what can be defined as “home sperm freezing”. While some make the donor complete the freezing at home, most of them include a collection vessel with a dilution solution that has to be shipped to a laboratory for freezing. Samples frozen with this kind of kit (i.e., cryopreserved after shipment) showed similar post-thawing parameters when compared to conventionally (in loco) frozen controls [29] and maintained sufficient motility to be used for assisted reproduction techniques [30].

The present study aimed to assess whether a similar system could be used for dogs, performing semen collection and shipment directly from the field using a Styrofoam transport box cooled by two icepacks. However, something that would need to be considered also before applying this system for banking or breeding purposes is a suitable system for the identification of the donor animals (e.g., a veterinarian reading the donor dog’s microchip at the collection site) and a box sealing strategy to ensure that the sample arrives undamaged and unsubstituted to the freezing facility.

Although the freezability of dog semen after prolonged chilling was evaluated in the past in laboratory conditions [12,13,14,15] or using the same transport box used in the present study after equilibration in a refrigerator [16], the present work is the first one where collection has been performed directly in the field, and the semen was diluted and placed in the transport box in loco without any laboratory equipment. A decrease in temperature is useful to slow down cell metabolism and prolong sperm cell viability, but field chilling without temperature control would not be ideal to maintain sperm quality before freezing. Instead, the results obtained in the present in-field study were encouraging, since they were comparable to those obtained in laboratory settings, where semen could be kept stably cool for up to 48 h before freezing [12,13].

In more detail, the only parameter that tended to decrease along time, even if not significantly, was motility. Therefore, it would be desirable to ensure that the transport takes place in the shortest time possible, ideally in less than 24 h, in order to have better post-thawing motility, comparable, in absolute value, to the samples conventionally frozen right after collection. Freezing after 48 h of transport remains an alternative where a faster shipment is not possible, but it must be taken into account that a greater number of spermatozoa will probably be required to reach the inseminating dose, which is usually calculated based on motile spermatozoa (for transcervical insemination, 150–200 million live, morphologically normal spermatozoa for the best pregnancy rates [31]).

It is known that the most evident effect of spermatozoa cryopreservation is a decrease in motility [32,33], and that there is a sharp decrease during the post-thawing time [12], as was also noticeable in the present results of motility 6 h post-thawing. This could be explained by the fact that cryopreserved spermatozoa undergo precocious capacitation [34], which can occur as early as in 2 h for frozen spermatozoa, compared to 4 h for fresh samples in vitro [35]. Indeed, capacitation determines a hyperactivation of motility, which reduces the spermatozoa survival [36].

Morphological parameters (MAI, TZI, SDI, acrosome, and membrane integrity) remained similar regardless of freezing time, but as expected, all significantly lower than the fresh control. Frequent morphological anomalies in the frozen samples were acrosomal defects, which contributed to the increase in the WHO morphological indices, especially SDI, which counts all the head defects. This trend was confirmed by the FITC-PNA/PI staining and by lower membrane integrity. Cryopreservation often alters the phospholipid bilayer of cell membranes, and such damaged membranes contribute to cause both spermatozoa capacitation and early acrosome reaction.

Extenders (chilling extender or freezing extender 1, both lab-made according to [3]) had no influence on any of the parameters hereby assessed. They were similar in composition, but the freezing extender 1 contained 3% glycerol. Although cryoprotectants can be toxic, this concentration is the one usually employed in the first step of Uppsala freezing, and it did not affect the spermatozoa’s post-warming quality also when used for a prolonged time (i.e., 24 or 48 h). In less specialized settings, such as in small practices where semen dilution for freezing is based on the ejaculated volume, as suggested by some producers of commercial extenders, the use of the freezing extender 1 as a holding medium for semen transport before freezing could be useful to simplify the cryopreservation procedure. Indeed, instead of counting and re-diluting the spermatozoa, freezing extender 2 could just be added in the same amount as extender 1, without the need of semen centrifugation, the effect of which deserves to be investigated [16]. Other diluents or the addition of supplements with putative ameliorative effects could also be evaluated to enhance pre-freezing and post-thawing semen quality. For instance, since oxidative stress is an issue both during cooling and cryopreservation [11,24,37,38], the addition of antioxidants could be useful to limit cellular damages [15].

Although further in vivo studies would be needed to assess the fertilizing ability of semen cryopreserved after cooled transport, the present study was the first one to assess its in vitro fertilizing ability thanks to the ZBA, revealing no differences between fresh and frozen semen, between different freezing times, and between different extenders. Since cryopreservation damaged cell membranes and acrosomes, this could affect spermatozoa adhesion capacity to the zona pellucida. The present results could be due to effective maintenance of the ability to adhere to the zona pellucida or to the intrinsic limits of the ZBA such as the binding capacity of spermatozoa without an intact acrosome [39] and the variability of the frozen ovaries-derived oocytes [23]. Nevertheless, the usefulness of this test is widely recognized and even suggested [12] for studies such as the one presented here. More standardized tests could be applied in future experiments to confirm the possibility of freezing semen samples after cooled transport, such as the hemizona assay, which allows for the direct comparison of multiple samples (usually two) using different portions of the same zona pellucida [40].

Dogs of different breeds, sizes, and ages were included in this study, meaning important biological variability in the fresh semen quality, derived from, among other factors, age and body size, will obviously influence the post-thaw quality. However, it was important to have an inhomogeneous dog population to assess whether a tendency could be found. Indeed, the results could show a common thread, which was that the pre-freezing storage time should be kept shorter. High standards deviations together with the number of dogs included in the study is something to consider when evaluating the results of the present study. Pooling of ejaculates from different dogs would have decreased the variability, but it would have not been representative of the actual reality, where semen is collected from a single subject and individually cryopreserved. The same applies to wild canids, where individual variability also exists and which could also benefit from a semen preservation protocol such as the one proposed in this study.

## 5. Conclusions

Freezing of dog-ejaculated semen could be performed after holding in an icepack-cooled transport box for up to 48 h without affecting spermatozoa morphofunctional features. Shorter transport time (24 h) is advisable to better maintain motility, otherwise this protocol could only be applied to samples with a high number of spermatozoa that would provide an inseminating dose even when motility is low. Subject variability as well as dog identification issues should also be considered before this system can be applied for routine semen cryopreservation and dog breeding.

## Figures and Tables

**Table 1:** Morphological features of ejaculated canine spermatozoa at collection (Fresh), after standard freezing (Frozen@0h), or freezing at different times of storage in a transport box (Frozen@24h and Frozen@48h).

	MAI	TZI	SDI	Intact Acrosomes (%)	Intact Membranes (%)
Fresh	1.16 ± 0.28 ^a^	0.99 ± 0.42 ^a^	0.43 ± 0.28 ^a^	89.91 ± 10.51 ^a^	89.87 ± 7.67 ^a^
Frozen@0h	1.59 ± 0.11 ^b^	1.51 ± 0.11 ^b^	1.48 ± 0.18 ^b^	52.27 ± 18.22 ^b^	65.37 ± 17.44 ^b^
Frozen@24h	1.61 ± 0.26 ^b^	1.56 ± 0.24 ^b^	1.52 ± 0.28 ^b^	42.1 ± 25.22 ^b^	62.41 ± 21.69 ^b^
Frozen@48h	1.55 ± 0.23 ^b^	1.48 ± 0.18 ^b^	1.47 ± 0.24 ^b^	51.1 ± 16.86 ^b^	62.02 ± 16.05 ^b^

^a, b^ Indicate significant differences within columns (*p* < 0.05). MAI = multiple abnormalities index; TZI = teratozoospermic index; SDI = sperm deformity index.

**Table 2:** Subjective motility of ejaculated canine spermatozoa after standard freezing (Frozen@0h) or freezing at different times of storage in a transport box (Frozen@24h and Frozen@48h).

	Motility Pre-Freezing (%)	Motility at Thawing (%)	Motility 6 h Post-Thawing (%)
Frozen@0h	86 ± 13.5 ^a^	45.5 ± 19.5 ^a^	11.3 ± 10.41 ^a^
Frozen@24h	73 ± 17.67 ^a, b^	41.3 ± 23.69 ^a^	8.3 ± 15.15 ^a^
Frozen@48h	58.1 ± 23.68 ^b^	27 ± 18.74 ^a^	4.78 ± 4.32 ^a^

^a, b^ Indicate significant differences within columns (*p* < 0.05).

**Table 3:** Ability of ejaculated canine spermatozoa to bind homologous zonae pellucidae at collection (Fresh), after standard freezing (Frozen@0h), or freezing at different times of storage in a transport box (Frozen@24h and Frozen@48h).

	Spermatozoa Per Oocyte (*n*)	Spermatozoa Per “Bound Oocyte” ^1^ (*n*)	Oocytes with Bound Spermatozoa (%)
Fresh	9.18 ± 7.93	30.05 ± 60.17	56.57 ± 33.57
Frozen@0h	7.77 ± 10.02	10.96 ± 11.71	55.5 ± 30.95
Frozen@24h	7.12 ± 10.19	10.13 ± 13.73	48.11 ± 28.43
Frozen@48h	10.71 ± 9.2	13.68 ± 9.05	64.44 ± 33.41

No significant differences within columns (*p* > 0.05). ^1^ Bound oocyte = oocyte with at least one spermatozoon bound to its zona pellucida.

**Table 4:** Post-thaw morphological features of ejaculated canine spermatozoa after dilution with chilling (C) or freezing extender 1 (F), chilling and storage for 24 or 48 h in a transport box, and Uppsala freezing.

	MAI	TZI	SDI	Intact Acrosomes (%)	Intact Membranes (%)
Frozen@24h-C	1.4 ± 0.36	1.30 ± 0.28	1.03 ± 0.6	62.77 ± 20.26	54.48 ± 14.95
Frozen@24h-F	1.43 ± 0.29	1.33 ± 0.32	1.12 ± 0.54	67.62 ± 18.13	57.92 ± 12.72
Frozen@48h-C	1.48 ± 0.26	1.39 ± 0.2	1.37 ± 0.34	63.29 ± 21.66	50.78 ± 19.38
Frozen@48h-F	1.48 ± 0.17	1.38 ± 0.13	1.35 ± 0.25	59.97 ± 12.68	58.66 ± 14.09

No significant differences within columns (*p* > 0.05). MAI = multiple abnormalities index; TZI = teratozoospermic index; SDI = sperm deformity index.

**Table 5:** Subjective motility of ejaculated canine spermatozoa after dilution with chilling (C) or freezing extender 1 (F), chilling and storage for 24 or 48 h in a transport box, and Uppsala freezing.

	Motility Pre-Freezing (%)	Motility at Thawing (%)	Motility 6 h Post-Thawing (%)
Frozen@24h-C	69 ± 21.83 ^a^	47 ± 16.36 ^a^	24.9 ± 23.67 ^a^
Frozen@24h-F	68 ± 18.74 ^a^	44 ± 21.19 ^a, b^	26.2 ± 20.79 ^a^
Frozen@48h-C	57.1 ± 28.47 ^a^	23 ± 15.49 ^b^	8.44 ± 12.42 ^a^
Frozen@48h-F	54.1 ± 24.79 ^a^	33.6 ± 21.44 ^a, b^	10.22 ± 10.97 ^a^

^a, b^ Indicate significant differences within columns (*p* < 0.05).

**Table 6:** Post-thaw ability of ejaculated canine spermatozoa to bind homologous zonae pellucidae after dilution with chilling (C) or freezing extender 1 (F), chilling and storage for 24 or 48 h in a transport box, and Uppsala freezing.

	Spermatozoa Per Oocyte (*n*)	Spermatozoa Per “Bound Oocyte” ^1^ (*n*)	Oocytes with Bound Spermatozoa (%)
Frozen@24h-C	21.79 ± 22.82	24.79 ± 24.3	79.22 ± 18.76
Frozen@24h-F	7.28 ± 11.32	8.78 ± 11.84	55.75 ± 36.02
Frozen@48h-C	7.75 ± 6.22	9.85 ± 6.69	77.67 ± 27.53
Frozen@48h-F	11.91 ± 11.24	14.13 ± 11.23	75 ± 22.73

No significant differences within columns (*p* > 0.05). ^1^ Bound oocyte = oocyte with at least one spermatozoon bound to its zona pellucida.

## Data Availability

Data are contained within the article.
